# The black summer bushfires: impacts and risk factors for livestock bushfire injury in south‐eastern Australia

**DOI:** 10.1111/avj.13165

**Published:** 2022-05-05

**Authors:** BD Cowled, A Hillman, MP Ward, H Clutterbuck, M Doyle, J Webb Ware, M Thomas, K Plain, R Barwell, M Laurence, C Pfeiffer

**Affiliations:** ^1^ Ausvet Pty Ltd 34 Thynne St Bruce Australian Capital Territory Australia; ^2^ Sydney School of Veterinary Science, Faculty of Science University of Sydney 425 Werombi Road Camden New South Wales Australia; ^3^ South East Local Land Services 159 Auburn St Goulburn New South Wales Australia; ^4^ Melbourne Veterinary School, Faculty of Veterinary and Agricultural Science University of Melbourne Grattan St Parkville Victoria Australia; ^5^ Animal Health Australia Level 2, 95 Northbourne Ave Turner Australian Capital Territory Australia; ^6^ Meat and Livestock Australia Level 1, 40 Mount Street North Sydney New South Wales 2060 Australia

**Keywords:** Australia, bushfire, injury, livestock, risk factors, wildfire

## Abstract

**Background:**

The 2019/2020 Australian bushfires were the largest bushfire event in modern Australian history. While actions to mitigate risk to homes from bushfires are well reported, there is very little research reported on the impacts of bushfires on livestock. With an increasing incidence of bushfires predicted, there is an urgent need to identify how farmers can best protect their livestock.

**Objectives:**

Compare bushfire affected farms with and without injured livestock to identify associations between risk factors and bushfire injury. Infer management approaches that can be used to reduce bushfire injury in livestock.

**Method:**

A case‐control study using a structured interview questionnaire, delivered in late 2020 to cattle and sheep farmers in south‐eastern Australia (New South Wales and Victoria) whose farmland was burnt in the 2019/2020 Australian bushfires. Case farms were farms with bushfires injured or killed livestock. Control farms were farms that had no bushfire injured livestock but that still had fire present on the farm. Interview responses were summarised and information theoretical approaches were used to identify potential risk factors for livestock bushfire injury and protective actions that could inform future fire‐preparation recommendations.

**Results and discussion:**

Of 46 farms in the case‐control study, 21 (46%) reported bushfire injured or killed livestock. Apparent protective factors identified included: preparation (having a bushfire plan and more than two farm bushfire fighting units), backburning and receiving assistance from fire authorities. Combined beef and sheep grazing enterprises appeared to have an increased risk of bushfire injury to livestock.

Bushfires (also known as wildfires) are increasing in frequency globally, especially as a result of longer fire seasons in temperate or boreal regions.^1^, ^2^ South‐east Australia endured a severe drought preceding and including the 2019–2020 spring and summer. Then in the spring and summer, Australia experienced a severe bushfire event widespread across multiple states (Queensland, New South Wales [NSW], Victoria and South Australia). During this bushfire event more than 17 million hectares of land burnt, more than 3,000 homes were destroyed and 33 people died.^3^ It was the largest Australian bushfire event ever recorded^3^ and has become known as the ‘Black Summer Bushfires’.

It was estimated that more than 56,000 livestock were killed or euthanised in New South Wales, Victoria and South Australia, perhaps as many as 69,000.^4^, ^5^ However, large numbers of livestock are present in these areas. For example livestock population data^6^, ^7^ indicate that there were 3.6 million cattle and 21 million sheep in bushfire affected regions of NSW and Victoria (BC, unpublished data). This indicated the overall proportion of livestock killed by bushfires was relatively low. The real issue is that the impact on some individual producers was very high, with high stock losses on particular farms.

There is well‐developed literature examining risk mitigation and resilience from bushfires for infrastructure such as homes. For example, Calkin, Cohen, Finney and Thompson^8^ examined current knowledge including risk management and decision science to conclude how to protect homes from burning. Other authors examine prescribed burning specifically,^9^ insurance and mitigation or education campaigns^10^ and household preparedness and response.^11^

Contrastingly there is very little research conducted globally on the impacts of bushfires on livestock.^12^, ^13^, ^14^, ^15^, ^16^, ^17^, ^18^, ^19^, ^20^, ^21^, ^22^, ^23^, ^24^, ^25^ For example, a comprehensive literature review of bushfire and livestock literature by a subset of authors (BC, AH, MT and CP) identified 14 global publications, mostly case studies in Australia, but not including risk factor studies.^12^, ^13^, ^14^, ^15^, ^16^, ^17^, ^18^, ^19^, ^20^, ^21^, ^22^, ^23^, ^24^, ^25^ This limits understanding of protective measures which could assist in the protection of livestock from bushfires. That is, what steps can producers take to minimise the probability that livestock will be injured by fire, either through mitigation or preparation before a bushfire or in response after a fire has arrived on a farm.

The objectives of this study were:

1. Compare bushfire affected farms that had injured livestock with those without injured livestock to identify associations between risk factors and bushfire injury.
2. Infer management approaches that can be used to reduce bushfire injury in livestock when a farm is impacted by bushfire.

## Materials and methods

### Study design

The study was a case‐control study of farms in south‐east Australia that were affected by the 2019/2020 Australian bushfire emergency. All farms enrolled in the study had all or a portion of the farm burnt by bushfire in the 2019/2020 Australian bushfire season. Therefore, the study occurred at the level of the farm and not individual animals, although the number of livestock injured or killed per farm was recorded.

Case farms were farms with one or more bushfires injured or killed cattle or sheep. Injured animals included those with visible damage to feet, hooves, or skin, acute respiratory injury, but not those with singed hair/wool only, while killed animals included both those that perished in the face of the fire as well as those that were humanely destroyed with injuries in the aftermath – (see Cowled et al.^26^ for further description of the injuries observed). Control farms were farms that had no bushfire injured livestock but that still had fire present on the farm. Control farms included farms that could have had livestock with minor singes (e.g. burnt hair but no injury to living tissue).

The study was retrospective in that recruitment of farms occurred 6–9 months after the fires were extinguished and therefore cases were prevalent cases.

### Study area and population

**Figure 1:**
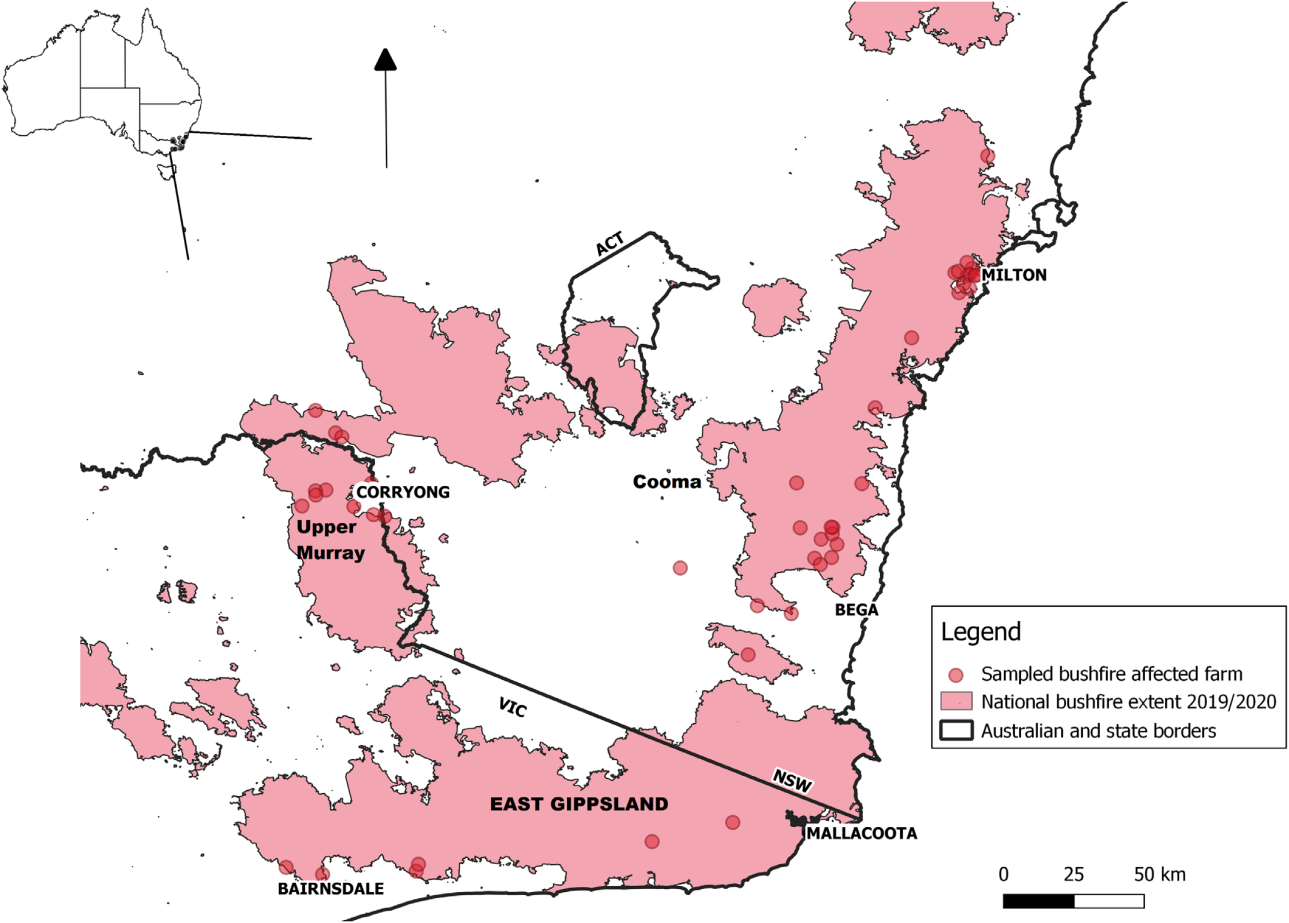
The location of sampled bushfire‐affected farms in South‐Eastern Australia.

This research purposively selected a geographic area from which to sample bushfire‐affected farms. This area was in the south‐east of New South Wales and northern and eastern Victoria and was the main bushfire‐affected region in south‐east Australia in November and December 2019 and January 2020. Thus, farms from two state jurisdictions were sampled. The sampled case and control farms are shown in Figure 1 with an overlying bushfire extent.

Farms with cattle (either dairy or beef) that had bushfires anywhere on the farm were eligible for inclusion in the study. Some of those cattle farms also had sheep and were, therefore, mixed sheep and cattle enterprises. However, the majority of the study area was a coastal area which meant that the majority of the burnt farms had cattle rather than sheep. Therefore sheep only farms (which did occur in some limited areas of our study site) were precluded to avoid confounding or analytical issues. Adding a small number of sheep farms that may have quite different risk factors or protective factors compared to the cattle farms that formed the majority of the study would not have resulted in a large enough sub‐sample size for meaningful interpretation.

### Selection of cases and controls

In NSW, the Local Land Services (LLS) District Veterinarians responsible for each target district (Bega, Berry/Milton and Cooma) were consulted in August 2020, approximately 7 months after the fires finished. Consultation attempted to identify all fire‐affected farms in their district, based on veterinarian recall and diary entries (recognising that all landholders located within a district are known to LLS authorities and recorded on a database). All fire‐affected farms known to have burnt livestock (cases) were collated and all were subsequently contacted to determine whether they would participate in a study. An additional equal sample of fire‐affected farms without bushfire injured livestock (controls) was randomly selected from the same district and was approached for inclusion as control farms. In effect, all fire‐affected landholdings in the region that contained livestock (beef and dairy cattle including some mixed sheep and beef farms) were eligible and the sampling frame is the list of farms held by NSW LLS in the South East of NSW.

A similar approach was taken in East Gippsland (Victoria) and the Upper Murray area (Vic‐NSW border), except that the private veterinarians rather than LLS district vets were consulted to identify their clients with burnt farms to provide cases and controls in October and November 2020 (9–10 months after fires finished)In the Upper Murray, fire‐affected farms with fire injured or killed livestock (cases) and an additional equal random sample of farms without bushfire injured livestock (controls) were contacted to enrol in the study. In East Gippsland, no candidate farms meeting the case criteria that were willing to participate were identified, so only control farms were recruited. Where initially selected farms declined to participate, some convenience sampling was required to recruit sufficient study farms.

Participants were provided with a plain language statement and initial contact was established by mail and/or telephone. All participating farms were visited to deliver a questionnaire interview. In addition, biological specimens and spatial mapping of the fire and impacts were collected for parallel studies.

In summary, the key features of the case‐control design were:

- Retrospective case‐control study
- Study base: farms in primary veterinary databases (LLS recorded farms in NSW, private veterinary databases in Victoria)
- Eligibility criteria for farms: Located in a bushfire‐affected region of South East Australia (NSW or Victoria) and bushfire burned on the farm sometime between November 2019 and February 2020. Containing cattle or both cattle and sheep.
- Exposure variables: Several factors including factors affecting fire intensity (e.g. vegetation, wind direction), risk mitigation activities (e.g. prescribed burning, preparation for fire season) and response to fire (e.g. active fire fighting, moving stock).
- Case definition: Fire‐affected farms with one or more bushfires injured livestock on the farm. All identified cases were approached for study entry.
- Control definition: Fire‐affected farms that did not have injured livestock. Approximately the same number of controls from each district/region as there were cases in that district/region.
- Risk‐based design: We assumed the population of farms was closed due to the short period of time over which fires occurred. Controls were selected from farms that did not become cases by February 2020.

### Data collection

A structured questionnaire was designed by several co‐authors (CP, BC, HC, JWW, MT). Questions were focused on possible risk factors for injured livestock and were identified through literature reviews, discussion with experts (e.g. firefighters) and the experience of the co‐authors. The questionnaire was piloted on a single farm, revised modestly and then deployed with the same questions asked to both the cases and controls. The questionnaire is included in the Supporting information.

The questionnaire was delivered face to face by a veterinarian in most cases, except for a small number (two controls) by phone or video conferencing due to COVID‐19 restrictions. The questionnaire was digitised in Qualtrics software (https://www.qualtrics.com) for computer‐aided delivery and recording of responses during all interviews, resulting in a digital database of all data collected during interviews. Interviews were conducted between July 2020 and January 2021.

Farmers were asked to answer most of the bushfire‐related questions considering the worst day of the fires. This was the day when they perceived the highest risk of damage from the bushfire if fires burned for multiple days on their farm. Only a small proportion of farms had bushfires for multiple days with most of them having fires on one day. The questionnaire is available in Supporting material.

This study focused on analysing a portion of the questionnaire data, those parts concerned with risk factors for livestock bushfire injuries (burns) or death. The analysis of fire recovery, farm impacts, livestock nutrition, biosecurity and animal health are presented in separate *fora*.

In addition, open‐source data representing potential confounders was sourced. In particular, data was sourced for the 2 months before the fire on monthly total pasture biomass, monthly relative pasture growth and monthly rainfall. This was downloaded from AussieGRASS, The Long Paddock (Department of Science, Information Technology and Innovation, 2015).^27^ This was analysed as a monthly average for the 2 months preceding November 2019, noting that fires began in November 2019 in the study area and minimal rainfall occurred across study areas subsequent to this date.

Spatial data from fire authorities representing the bushfire burnt area extent, dates and intensity were also identified, although this information relevant to each farm was also requested directly in the interview. In more detail, New South Wales 2018/2019 and 2019/2020 season bushfires data were obtained from the New South Wales Rural Fire Service (RFS), provided as a shapefile. Data on bushfires in Victoria were provided as a shapefile from the Victorian Department of Environment, Land, Water and Planning.

### Manipulation of variables

Dry stock equivalents (DSE) were calculated based on the relative DSE weights for various categories of sheep, beef and dairy cattle reported from each farm. The relative DSE weights for various stock classes were sourced from Agriculture Victoria resources.^28^

The wind direction reported by farmers and comments about wind by farmers were interpreted and reported as the main wind direction in the preceding 12 h of the worst time of the fire (if multiple days of fire were reported on a farm) or when fire arrived at the farm. The many wind directions were categorised into four main directions namely, westerly, easterly, northerly and southerly. For example, north‐westerly, westerly, south‐westerly and north‐north‐west wind directions were all recorded as westerly.

Speed of fire on pasture was missing in two respondents' data. The value of independent bushfire data from the data overlying the farm was used to generate a value, that is, the overlying fire records from the fire authorities (RFS) was used to give both values a ‘fast’ fire speed as the fire record indicated that it was a major fire, burning a very large area in a short period of time.

### Analysis

Several hypotheses were developed a priori that sought to explain the occurrence of farms with livestock with bushfire injury. These hypotheses are outlined in Table 1 including the model implemented to represent the hypotheses.

**Table 1:** The a priori hypotheses developed that sought to explain the prevalence of bushfire injury from the case‐control study comparing burnt farms with injured and uninjured livestock

Hypotheses	Explanatory variables	Model implemented	Explanation
Wind direction	The wind direction at the time the fire was at its worst or when it hit a farm (westerly influence, easterly influence, south, north)	$\log_{e}\left(\frac{p}{1-p}\right)=\beta_{0}+\beta_{1}\text{Wind direction}+\text{random effect}\;\left(\text{district}\right)$	Very strong wind from the west is associated with case farms as the fire is more severe
Intensity of fire	Speed of fire (fast or otherwise) Height of flames (m) Width of fire front (<400 m or ≥400 m) For both forest and pasture fires	$\log_{e}\left(\frac{p}{1-p}\right)=\beta_{0}+\beta_{1}\text{Speed}+\beta_{2}\text{Height}+\beta_{3}\text{Width of front}+\text{random effect}\;\left(\text{district}\right)$	Fire intensity is a function of speed, size of flames, and width of fire front
Vegetation removal	Grazed down refuge paddocks (yes/no) Remove large trees from pasture (yes/no)	$\log_{e}\left(\frac{p}{1-p}\right)=\beta_{0}+\beta_{1}\text{Refuge paddock}+\beta_{2}\text{Remove large tree}s$	A higher fuel load (less vegetation management) where stock is located is associated with case farms
Pasture biomass and recent rainfall	Monthly pasture biomass (kg dm/ha) in 2 months before November 2019 at farm Monthly total rainfall (mm) in 2 months before November 2019 at farm	$\log_{e}\left(\frac{p}{1-p}\right)=\beta_{0}+\beta_{1}\text{Pasture biomass}+\beta_{2}\text{Rainfall}+\beta_{3}\text{Rainfall}:\text{Pasture biomass}$	Higher pasture volume, especially if dry is associated with case farms due to additional fuel
Preparation for fire (planning)	How many fire fighting units did you have? (0, 1–2, >2) Did you have a fire‐plan in place (yes/no)?	$\log_{e}\left(\frac{p}{1-p}\right)=\beta_{0}+\beta_{1}\text{Fire fighting units}+\beta_{2}\text{Fireplan}$	The preparation the producer made to mitigate the fire was associated with being a control farm
Response to fire (move stock)	Did you move stock within farm in response to fire (yes/no)? Did you move stock from the farm in response to fire (yes/no)?	$\log_{e}\left(\frac{p}{1-p}\right)=\beta_{0}+\beta_{1}\text{Move stock from farm}+\beta_{2}\text{Move stock}\;\mathrm{on}\;\text{farm}$	Moving stock away from the path of the fire was associated with being a control farm
Response to fire (firebreak)	Did you install a firebreak (yes/no)? Did you move stock within farm in response to fire (yes/no)?	$\log_{e}\left(\frac{p}{1-p}\right)=\beta_{0}+\beta_{1}\text{Install}\;a\;\text{firebreak}+\beta_{2}\text{Move stock}\;\mathrm{on}\;\text{farm}$	Installing a firebreak to interrupt fire was associated with being a control farm, but the livestock may have had to be moved to take advantage of the firebreak
Response to fire (cut fences)	Did you cut fences to enable stock to escape fire (yes/no)?	$\log_{e}\left(\frac{p}{1-p}\right)=\beta_{0}+\beta_{1}\mathrm{Cut}\;\text{fences}$	Cutting fences to enable stock to escape fire was associated with being a control farm
Response to fire (stay and defend and number of firefighters)	Did you stay and defend (yes/no)? How many fire‐fighting staff (0, 1, 2–3, 4–5, 6+)	$\log_{e}\left(\frac{p}{1-p}\right)=\beta_{0}+\beta_{1}\text{Stay and defend}+\beta_{2}\mathrm{how}\;\text{many fire fighting staff}$	Staying and defending with enough staff was associated with being a control farm
Response to fire (reliance on government fire authorities)	Did you plan on country fire authority or rural fire service to fight the fire (yes/no)? Did you receive assistance from CFA or RFS (yes/no)?	$\log_{e}\left(\frac{p}{1-p}\right)=\beta_{0}+\beta_{1}\text{Plan}\;\mathrm{on}\;\mathrm{CFA}\;\text{or}\;\mathrm{RFS}+\beta_{2}\text{Receive}\;\mathrm{RFS}\;\text{or}\;\mathrm{CFA}\;\text{assistance}$	State government bushfire fighting authorities' assistance (RFS and CFA) allowed effective fire fighting and was associated with being a control farm
Fire fighting activities (backburning)	Did you backburn during the fire (yes/no)	$\log_{e}\left(\frac{p}{1-p}\right)=\beta_{0}+\beta_{1}\text{Backburn}$	Lighting a backburn to consume fuel and assist management of the bushfire was associated with being a control farm
Fire fighting activities (containment lines)	Did you establish containment lines during the fire (yes/no)	$\log_{e}\left(\frac{p}{1-p}\right)=\beta_{0}+\beta_{1}\text{Containment line}$	Establishing containment lines around a bushfire assists management of the bushfire was associated with being a control farm
Fire fighting activities (fighting fire with water)	Did you fight fires with water during the fire (yes/no)	$\log_{e}\left(\frac{p}{1-p}\right)=\beta_{0}+\beta_{1}\text{Fight with water}$	Fighting fire with water to assist management of the bushfire was associated with being a control farm
Production type	Production type (beef cattle, beef cattle and sheep, dairy cattle), including land area (ha) of farm and the number of dry stock equivalents	$\log_{e}\left(\frac{p}{1-p}\right)=\beta_{0}+\beta_{1}\text{Prod Type}+\beta_{2}\text{Area}+\beta_{3}\mathrm{DSE}$	The production animal being farmed is more or less susceptible to fire injury and this is confounded by stocking rates (area and DSE)
Management of stock	Grazing strategy (set stocking, rotational grazing or both) Stocking rate perception (conservative, medium and high)	$\log_{e}\left(\frac{p}{1-p}\right)=\beta_{0}+\beta_{1}P\text{Grazing strategy}+\beta_{2}\text{stocking rate perception}$	Grazing management of livestock affects fuel load and set stocking and high stocking rate farms are associated with being a control farm

A simple or mixed logistic regression model that modelled the presence and absence of bushfire injured or killed livestock per farm against variables was implemented for each hypothesis. The random effect term represented the district in which the farm was found. The choice between a mixed and simple model for each hypothesis was made according to whether the use of a random effect term changed the standard errors of any other coefficients significantly. Care was taken to minimise the number of variables in each model, as the data set was not large, limiting the power to examine many variables concurrently in a single model.

The relative support for each hypothesis was examined using the information theory approach of Burnham and Anderson.^29^ Conditional model averaging occurred to estimate parameters across all models.^29^ Model averaging enables a weighting to be applied to each parameter depending upon the support the model has and which parameters are in which model. Inferences from the study were based upon these results, namely which hypotheses (represented by models) had empirical support (or were ranked highest) and how important were the variables comprising these hypotheses (based on the parameters predicted by model averaging).

In more detail, consistent with modern statistical approaches we did not rely upon null hypotheses testing^26^ or an arbitrary probability of less than 0.05^30^ to make decisions about the importance of a variable of interest as a risk factor. Instead, we first determined highly supported hypotheses using information theory.^29^ We determined a hypothesis had support from the data if its bias‐corrected Akaike Information Criterion (AICc Δ) difference (compared with the most supported model) was less than 2.^26^ We then examined explanatory variables in the supported models, examined P values and then performed exploratory post hoc modelling using identified variables.

Post hoc modelling happened using variables from the most supported models to attempt to identify a single model that best explained the information in the data. This comprised exploratory analyses and was not relied upon for model inferences.^26^ We examined the highly ranked hypotheses to determine the direction and size of the model averaged coefficients representing the variables of interest in the model. We used the highest P value of these coefficients (P = 0.11) as a cut point to include other variables of interest that were not in the supported models. That is, we identified the size, direction and significance (probability) of other model averaged co‐efficient to identify other variables that may have been important but were not included in supported models.

We did not use the post hoc modelling (including P values) to assess the importance of a risk factor, but rather to better understand the relationships of the variables and to allow insights to future research or further interpretation of the data, for example, the degree of variability in the outcome that appeared to be explained by the risk factors identified.

All analyses were undertaken in R statistical software (version 4.0.0) (see https://www.r-project.org/).

## Results

### Basic descriptive statistics

#### Response rate

Around 87 farmers from burnt farms were invited to participate in the study. Of this, 46 completed the survey; therefore, there was a response rate of 53%.

#### Demography of respondents

There were eight female and 37 male respondents (one preferred not to say). The median age of respondents was 60 years old (range: 30–79). Females tended to be younger with a median age of 54 years old (range: 30–71) than males with a median age of 61 years old (range: 35–79).

#### Farm characteristics, outcome variables and comparability of cases and controls

**Figure 2:**
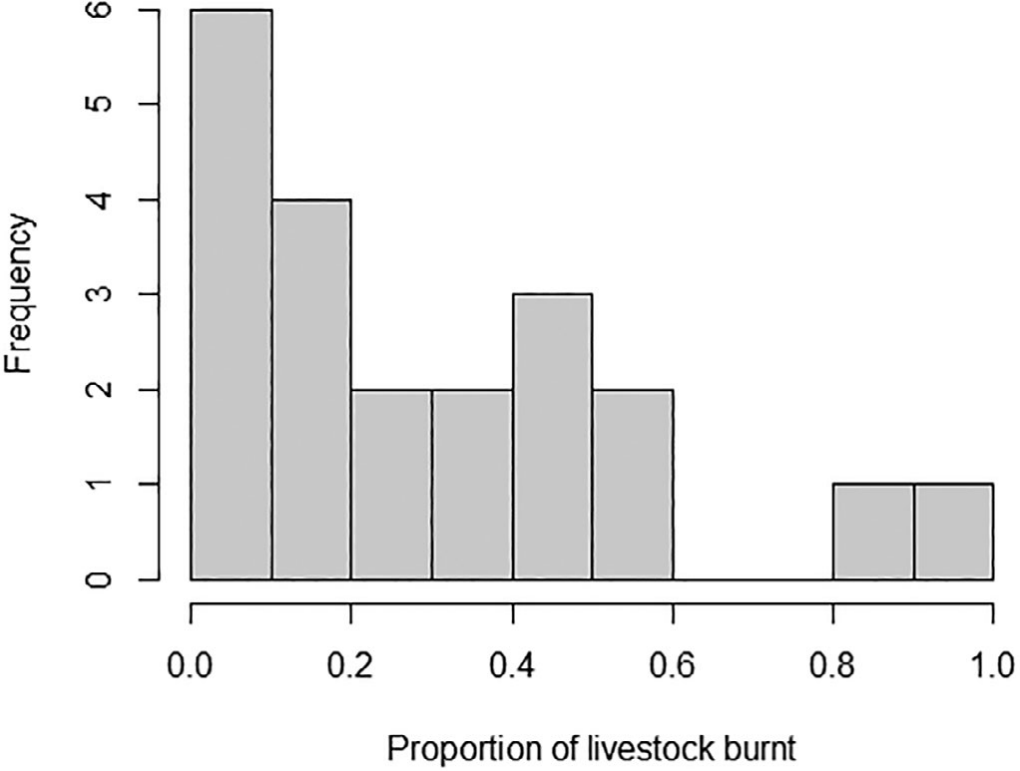
The proportion of individual livestock killed or injured per case farm. The proportion of livestock killed or injured per farm varied from 0% to 100%, with a median proportion killed of 20% (Q1–Q3: 8–43).

There were 46 farms in the case‐control study and 21 farms (46%) had bushfires that injured or killed livestock (see Table 2) meeting the criteria for a case farm. Of these farm categories with bushfire injured or killed livestock, 17 had burnt beef cattle, 6 had burnt sheep and one dairy had burnt cattle (see Table 2). The proportion of livestock killed or injured per farm varied from 0% to 100%, with a median proportion killed of 20% (IQR: 8–43) (see Table 3). See Figure 2 for the distribution. In terms of raw numbers of burnt or injured livestock, there was a median of 20 (IQR: 4–55) burnt beef cattle per farm, 90 (IQR: 40–265) sheep and 15 dairy cattle.

**Table 2:** The number of burnt farms (cases and controls) recruited into the study and the proportion of case farms by enterprise type

Farm enterprise type	Number of farms in the study	Number of farms with bushfire injured livestock	Proportion of enterprises with bushfire injured livestock (%)
Beef cattle	32	12	38
Beef cattle and sheep	9	8^a^	89
Dairy	5	1	20
Total farms	46	Total cases	21

^a^ 5/9 had cattle burnt and 6/9 had sheep burnt.

**Table 3:** Case farms divided by livestock enterprise type and proportion of individual livestock burnt per farm

Farm enterprise type with burnt livestock	Number of farms burnt livestock	Median proportion (%) of individual cattle burnt on a farm (Q1–Q3)	Median proportion (%) of individual sheep burnt on a farm (Q1–Q3)
Beef cattle only	12	25 (10–51)	‐
Beef cattle and sheep	8	8 (1–37)	12 (8–36)
Dairy	1	1 (−)	‐

Farm sizes varied from small non‐commercial farms of 16 ha and 102 DSE to larger farms up to 2,200 ha and 31,238 DSE. Farms were broadly comparable between cases and controls in terms of size and livestock numbers (DSE), although case farms had higher costs associated with bushfire damage (see Table 4). Total bushfire costs per farm ranged up to AUD 2,000,000, with a median cost of AUD 550,000 for case farms and AUD 100,000 for control farms. Rainfall, pasture growth and pasture biomass in the preceding 2 months were similar between cases and controls. See Figures S1–S3 in supplementary material for further information. Farms were located in south‐east Australia across five study districts (see Table 5 and Figure 1).

**Table 4:** Comparison of some key variables between case and control farms

Variable	Farms with burnt livestock (median, Q1–Q3)	Farms with no burnt livestock (median, Q1–Q3)
Financial damage due to bushfire (AUD)	AUD 550,000 (300,000–1100,000)	AUD 100,000 (45,000–220,000)
Farm size (ha)	243 (145–397)	206 (65–243)
Number of DSE on farm	1,231 (648–2,514)	877 (450–2,830)

**Table 5:** The location of sampled case and control farms and proportion of farms from each district

District where farm is located	Number of farms in the study	Number of farms with bushfire injured livestock	Proportion of farms with bushfire injured livestock (%)
Bega	16	8	50
Bombala	1	0	0
East Gippsland	5	1	20
Milton	12	5	42
Upper Murray	11	7	64

There were several interesting relationships identified between cases and controls and uncontrolled independent variables. For example, there appeared to be associations (possibly confounded) between several variables and being a case, including farm enterprise type, removal of woody vegetation, refuge paddocks, fire planning, the number of fire‐fighting units and backburning as fires approached. These are explored as two by n contingency tables in supporting information in Table S1.

### Modelling

#### A priori modelling

Three models (hypotheses) had empirical support in the data. In order, these were: preparation for fire (bushfire plan and the number of fire units), farm production type, fire‐fighting activities (backburning).

See the ranking and support for the various models in Table 6 with an AICc difference (Δ) of less than 2 indicating some support for the model.

**Table 6:** Akaike information criterion (AIC) values and other model selection metrics for bushfire models using information‐theoretic approaches^29^

Model/hypothesis	Degrees of freedom	Bias corrected AIC (AICc)	AICc difference (Δ)	Probability (Akaike weight)
Preparation for fire (planning)	4	63	0.00	0.277
Production type	5	63.3	0.39	0.228
Fire fighting activities (backburning)	2	64.1	1.11	0.159
Wind direction	4	65.1	2.15	0.095
Response to fire (reliance on fire authorities)	3	65.8	2.82	0.068
Vegetation removal	3	66.8	3.88	0.04
Fire fighting activities (containment lines)	2	67.3	4.37	0.031
Response to fire (cut fences)	2	67.6	4.65	0.027
Fire fighting activities (fighting fire with water)	2	67.7	4.75	0.026
Response to fire (firebreak)	3	69.1	6.13	0.013
Response to fire (move stock)	3	69.2	6.22	0.012
Intensity of fire in woodland	4	70.2	7.27	0.007
Pasture biomass and recent rainfall	4	70.8	7.9	0.005
Intensity of fire on pasture	4	71.6	8.7	0.004
Response to fire (defend and no. of firefighters)	5	72.4	9.45	0.002
Management of stock (grazing practices)	5	73.1	10.13	0.002

Models are presented in descending rank order from most supported to least supported. Models with a Δ of less than approximately two have substantial support. Therefore models above wind direction or response to fire all explain a substantial portion of the information in the data. Other models did not explain a significant part of the information in the data.

Variables from these supported models and selection of additional variables from the conditional model averaging revealed several variables that appear to be associated with the bushfire injury, either as protective or risk factors (see Table 7).

**Table 7:** Parameter estimates estimated using conditional model averaging across all models included in the a priori modelling

	Estimate	Odds ratio (95% CI)	Adjusted SE	Probability
(Intercept)	0.36	1.42 (0.15–13.67)	1.16	0.75
**Fire‐plan in place: Yes cf. baseline No**	**−1.78**	0.17 (0.03–1.11)	**0.96**	**0.06**
How many fire units: 1–2 cf. none	−0.28	0.76 (0.17–3.39)	0.76	0.72
**How many fire units: More than 2 cf. none**	**−2.29**	0.10 (0.01–1.34)	**1.32**	**0.08**
**Enterprise type: Beef cattle and sheep cf. baseline dairy**	**2.37**	10.67 (1.08–105.59)	**1.17**	**0.04**
Enterprise type: Beef cattle cf. baseline dairy	−2.11	0.12 (0.00–5.05)	1.90	0.27
Total DSE	0.00	1.00 (1.00–1.00)	0.00	0.24
Land area (ha)	0.00	1.00(1.00–1.01)	0.00	0.56
**Backburning?: Yes cf. baseline no**	**−1.84**	0.16 (0.02–1.53)	**1.16**	**0.11**
Wind direction: North cf. easterly influence	17.57	42712407.08 (0‐Inf)	2352.00	0.99
Wind direction: South cf. easterly influence	−17.57	0.00 (0‐Inf)	2880.00	1.00
Wind direction: Westerly influence cf. easterly influence	−0.29	0.75 (0.13–4.47)	0.91	0.75
Did you rely on fire authorities: Yes cf. no	1.17	3.21 (0.69–14.90)	0.78	0.14
**Did you receive assistance from fire authorities: Yes cf. no**	**−1.52**	0.22 (0.04–1.23)	**0.88**	**0.08**
Did you graze down refuge paddocks to shelter stock: Yes cf. no	−0.87	0.42 (0.10–1.79)	0.74	0.24
Do you routinely remove trees: Yes cf. no	−0.62	0.54 (0.12–2.35)	0.75	0.41
Did you install containment lines after fire: Yes cf. no	−0.41	0.66 (0.17–2.55)	0.69	0.55
Did you cut fences: Yes cf. no	−0.22	0.80 (0.20–3.16)	0.70	0.75
Did you attack fire with water: Yes cf. no	0.01	1.01 (0.25–4.09)	0.71	0.99
Did you install firebreaks: Yes cf. no	−0.57	0.56 (0.17–1.91)	0.62	0.36
Did you move stock on farm to protect them from fire: Yes cf. no	−0.11	0.90 (0.23–3.47)	0.69	0.88
Did you move stock from farm to protect them: Yes cf. no	−1.03	0.36 (0.03–4.07)	1.24	0.41
Speed of fire in woodland: Medium Cf. fast	0.38	1.47 (0.28–7.67)	0.84	0.65
Width of main fire front: <400 m cf. >400 m	−0.52	0.60 (0.14–2.59)	0.75	0.49
Height of fire in woodland (m)	−0.02	0.98 (0.93–1.03)	0.03	0.51
Average monthly rainfall in preceding 2 months (mm)	−0.02	0.98 (0.92–1.04)	0.03	0.47
Average monthly biomass in preceding 2 months (kg/ha)	0.00	1.00 (0.98–1.02)	0.01	0.86
Monthly rainfall (mm):Monthly pasture biomass (interaction)	0.00	1.00 (1.00–1.00)	0.00	0.90
Height of fire on pasture (m)	0.10	1.10 (0.67–1.81)	0.25	0.70
Speed of fire on pasture: Medium cf. fast	−0.08	0.92 (0.22–3.80)	0.72	0.91
How many fire fighting personnel: 2–5 cf. none	−0.03	0.97 (0.18–5.12)	0.85	0.97
How many fire fighting personnel: 6+ cf. none	−1.23	0.29 (0.02–3.63)	1.29	0.34
How many fire fighting personnel: 1 cf. none	0.85	2.33 (0.29–18.65)	1.06	0.42
Did you stay and defend: Yes cf. no	0.03	1.03 (0.28–3.76)	0.66	0.97
Grazing strategy: Rotational cf. combined set and rotational	0.74	2.09 (0.32–13.60)	0.96	0.44
Grazing strategy: Set cf. combined set and rotational	0.83	2.28 (0.27–19.73)	1.10	0.45
Perception of stocking rate: High cf. low	0.03	1.03 (0.22–4.80)	0.79	0.97
Perception of stocking rate medium cf. low	0.61	1.83 (0.43–7.80)	0.74	0.41

The bolded rows include coefficients that have a probability value of less than or equal to 0.11, which was the highest P value of coefficients in supported models. This was used to identify variables for post hoc explanatory analyse. Variables of interest include that protective effects were associated with having a fire plan in place before the fire, having more than two fire fighting units, being a dairy or beef cattle property compared with a mixed beef and sheep farm, backburning and receiving assistance from fire authorities.

The risk factors indicate that preparation including having several fire‐fighting units and a fire plan in place was protective. When a fire was occurring, having fire authorities present and having backburn lit (with or without fire authorities implementing the back burn) was protective. Being a combined sheep and cattle enterprise was a risk factor for bushfire injury.

#### Post hoc (exploratory) modelling

A single multifactorial model was established by incorporating all identified variables of importance (risk or protective) using the explanatory variables from supported a priori models into a single model. This produced the most informative model (AICc = 58.9) compared with the a priori models (Table 7, where the lowest AICc = 63). This model is presented in Table 8. Random effects for districts did not influence standard errors for other coefficients and were discarded from the model.

**Table 8:** Parameter estimates from the post hoc model incorporating the most important variables from the supported models assessed in a priori models

	Estimate	Odds ratio (95% CI)	SE	Probability
(Intercept)	2.84	17.13 (1.03–843.54)	1.67	0.09
Fire‐plan in place: Yes cf. baseline No	−2.41	0.09 (0.003–0.91)	1.34	0.07
Backburning?: Yes cf. baseline No	−2.25	0.11 (0.02–1.27)	1.50	0.13
How many fire units: 1–2 cf. baseline None	−0.47	0.62 (0.07–4.57)	1.04	0.65
How many fire units: More than 2 cf. baseline None	−2.85	0.06 (0.001–1.10)	1.71	0.10
Did you receive assistance from fire authorities: Yes cf. No	−2.10	0.12 (0.01–1.02)	1.23	0.09
Enterprise type: Beef cattle and sheep cf. baseline beef	2.90	18.14 (1.82–624.68)	1.40	0.04
Enterprise type: Dairy cf. baseline beef	−0.36	0.70 (0.03–8.95)	1.34	0.79

**Figure 3:**
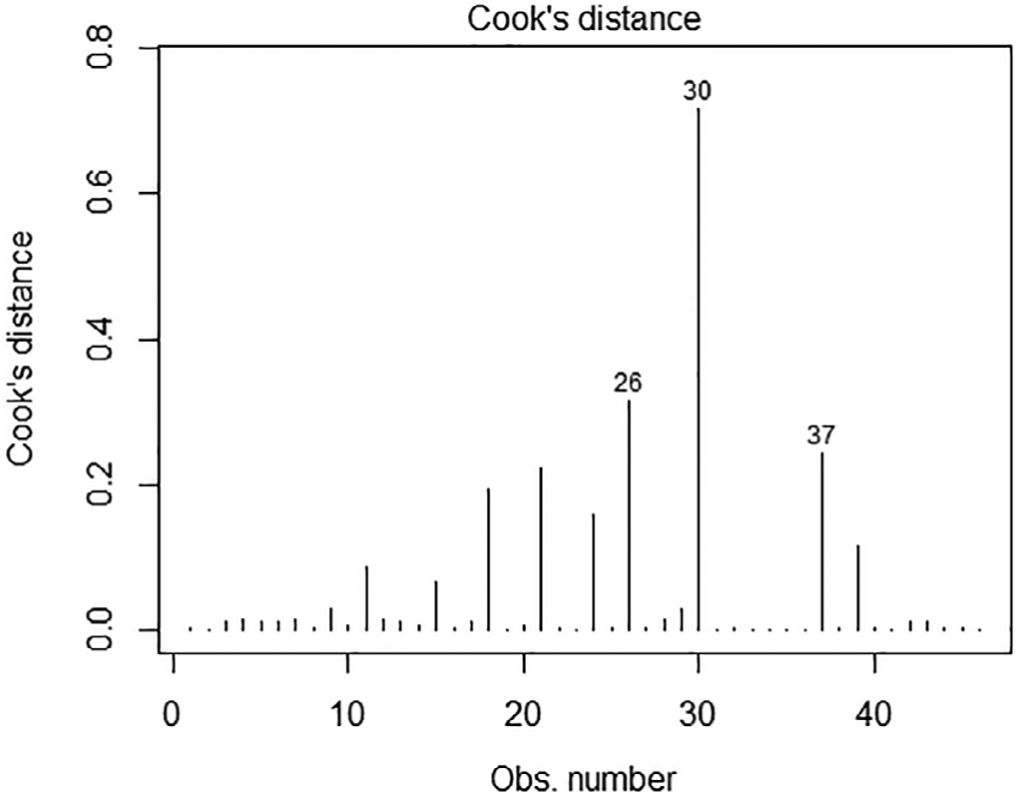
Cooke's distance for the multifactorial model. The figure indicates that there were several extreme observations (26, 30, and 37). These were manually checked and retained in the model.

Model fit for the multifactorial model was moderate. The pseudo r^2^ value was 38% indicating a 38% improvement in the log‐likelihood function due to the explanatory variables included in the model, compared with the model with no explanatory variables (an ‘intercept only’ model). A likelihood ratio x^2^ statistic comparing the multifactorial and intercept‐only model was significant indicating the multifactorial model provided a significantly better fit to the data than the intercept‐only model (x^2^ = 48.77, df = 7, P < 0.0005). Variance inflation factors were less than 1.5 for all variables indicating minimal or absent collinearity. Residuals were examined. A normal Q‐Q plot was produced and indicated that although there was linearity along most of the length of the plot there were some heavy tails indicating some extreme values (data not shown). Cooke's distance^31^ was plotted revealing that there were three extreme observations (Figure 3). These observations were checked and determined to be accurate and retained in the model. The observations that were extreme had two features. They could be farms that had burnt livestock but that had implemented all the protective factors that protected other farms on average (for example burnt stock but they had a fire plan, multiple fire units, back burnt, etc.). Or they were farms that had risk factors (e.g. combined beef and sheep farm) but did not have burnt livestock.

## Discussion

The 2019/2020 Australian bushfires were the largest recorded bushfire event in Australian modern history^3^ and had a severe and devastating impact on many farms. This study included farms with financial losses and costs up to AUD 2 million and with some participating farms experiencing up to 100% livestock losses. With an increasing incidence of bushfires predicted and recognition of the emerging effects of climate change,^32^, ^33^ there is a need to identify actions farmers can take to protect livestock. To that end, this research identified some risk factors that were associated with a higher probability of livestock bushfire injury or death. Whilst associations identified in observational studies cannot demonstrate causality, these results may be used to cautiously infer some protective actions that could be used to minimise livestock bushfire injury.

Planning in advance of the fire appeared to protect livestock from bushfire injury. In practical terms, this hypothesis was modelled by whether farmers reported they had a well‐thought‐out bushfire response plan and whether they had practical fire‐fighting equipment (namely mobile water tanks, hoses and pumps). It could therefore be inferred that producers who had thought, planned and equipped themselves adequately had an ability to reduce the probability of livestock from bushfire injury or death. This should be heartening for producers in future fires as it implies producers have agency in the face of severe fire. Other research has demonstrated that farmers often do have some form of bushfire plan (70%) even if it was not written and that many had independent water supplies and had cleared fuel from around houses.^22^

However, the knowledge that farmers can save livestock and other infrastructure should be cautiously applied. This is because the primary objective of farmers should be to protect the physical safety of people.^34^ There was a significant amount of variation in the outcome that was not explained by the best‐fitting post hoc model, which had a pseudo r^2^ value of 38%. There were also outliers in the data where farms had the protective factors in place, but livestock still died or was injured. Together this implies that there are other risk factors not modelled in our study or variation due to chance that can be associated with livestock injury and potentially human injury. In other words, even putting in place the protective risk factors for bushfire losses identified in this study (e.g. having good preparation with a farm fire plan and firefighting units, firefighting authorities support and back burns) does not always protect livestock. Therefore, consistent with fire authorities' advice, farmers who cannot be certain of their safety should evacuate to safer areas in the face of catastrophic fires,^35^ despite their responsibility to livestock and the probability of reducing the probability of livestock injury or death.

An additional protective factor was whether farmers implemented a backburn^36^ as the fire approached to consume available fuel and contain the fire. That is, the back‐burning model was a highly supported model and the variable itself appeared associated with the lower probability of bushfire injury. It is interesting to note that backburns were associated with a lower probability of injury statistically independently of fire authorities which indicates that farmers implemented backburns without fire authority involvement. This suggests that some sections of the farming community have expertise in fire management, as this is a complex and sometimes dangerous fire‐fighting tool. However, there are various legal impediments to implementing a backburn and caution must be applied when implementing such an action without the approval of firefighting authorities. Despite this, it suggests that fire authorities should engage with farmers to assist in the management of fires on farms and recognise the competence of this sector of the population in fire management.

As may be expected, the presence of a fire authority on a farm before or during the fire was protective. Whilst this was not a very well‐supported hypothesis (e.g. Δ > 2), the variable representing the provision of assistance by the RFS (NSW) or CFA (Vic) on a farm was associated with a lower probability of bushfire injury or death. This implies that the various actions implemented by fire authorities reduced damage on farms. While this assistance was protective, it was not always available in the 2019–2020 fire seasons due to the widespread nature and intensity of fire occurring across the study area. Therefore, this protective factor may be most relevant to localised fires where the threat to farm property is not geographically widespread and other approaches are likely needed to protect livestock where fire authority assistance is spread thin across the fire ground or focussed away from farmland.

In contrast, one of the most consistent and important risk factors for livestock injury was the type of livestock enterprise on a farm. Compared with dairy cattle farms and beef cattle farms, mixed beef and sheep farms were much more likely to have burnt livestock. It could be argued that this is because sheep are more prone to injury than cattle^37^ because their anatomy is different or because they are more difficult to move. However, there is some doubt as to this being an explanation in this study. For example, examining Table 3, it appears similar proportions of cattle and sheep were burnt on enterprises with both cattle and sheep, but that generally combined sheep/cattle farms were more likely to have at least one injured livestock than dairy farms or beef farms. It is uncertain what the explanation, may be, but it could be a variety of factors such as land type, the size of such farms, the ability of producers to manage two production species with different requirements in the face of approaching fire, or other variables. In addition, it is important to realise that whilst the probability of any injury was higher on combined beef and sheep farms, the proportion of cattle killed or injured on cattle only farms was generally higher than the proportion of cattle or sheep injured on combined farms once at least one injury occurred (see Table 3). This indicates a possible confounder was present in the study. Regardless this variable is not modifiable in advance of a fire and is probably less relevant as it cannot be manipulated to modify fire injury risk, even where it is causally associated with fire.

It is important to acknowledge the limitations of this study. These were principally associated with sample size and therefore statistical power. Despite being the largest study of its type worldwide to date, the sample size of 46 farms was relatively small in statistical terms. Whilst this size was sufficient to identify several protective and risk factors, the power may have been low for some variables where there may have been a relationship that was not as strong and therefore not detected (resulting in a type II error). Trauma, people leaving the industry and COVID‐19 were reasons that some people refused to participate.

As a result of the sample size, the absence of a statistically significant protective effect for some preparedness or response measures should be interpreted with caution. That is, the absence of a significant effect does not confirm that no protective effect for livestock occurs, but rather that these effects may be applied incompletely or sub‐optimally, modified by unmeasured confounders, or unable to be detected given the power constraints described above (type II error). For example, preparing a refuge paddock and moving stock there ahead of a fire is a frequently recommended protective strategy^38^, ^39^ but was not supported by the models presented (see Table 6 where the AICc Δ was 6.22 and Table 7, odds ratio 0.9, P value 0.88). This does not mean that moving stock has no protective effect as its effect may have been missed in our study. Likewise for other potentially useful actions such as vegetation removal from paddocks, managing pasture biomass, cutting fences and firefighting activities such as firebreaks or use of water to extinguish the fire. Modifiable explanatory variables that were not included in the post hoc model should not be discarded as ineffective based on this study alone. In contrast, the risk factors identified such as planning and backburns are likely to be accurately identified as useful as demonstrated through the highly ranked and supported models and variables with low P values.

Unfortunately, this sample size could not be increased in our study. Partly the study was limited by resources, in that we could not implement a national‐level study. However, the biggest limitation was the participation of farmers which was limited by several factors. Understandably, farmers experienced trauma associated with bushfire response and recovery on their farm and in their community including loss of human lives in some instances and some farmers did not wish to relive this trauma by participating in an interview. In addition, some landholders chose not to continue farming after the fires and were not available to be interviewed (Mark Doyle, LLS, Pers.com). The COVID‐19 pandemic limited interactions on farms between the veterinarians sampling and delivering questionnaires and farmers during the middle of the data collection phase. We were unable to collect data on non‐participating farmers and so are unable to estimate any biases that may have developed.

In the future, further understanding of bushfire preparedness and response and their relationship with livestock outcomes may be investigated with alternative methods such as qualitative research interviews and thematic analysis.^40^ Increasing the depth of data collected from a smaller sample of farms using this approach may be more feasible and contribute to an increasing evidence‐based. This would be useful after a bushfire where only small numbers of farmers may be available for interview and some convenience sampling may be required. However, the application of a case‐control study to a rare outcome such as bush fire injuries in livestock is also a useful means of investigation and revealed some important risk factors in this study.

Other limitations may be associated with other biases such as recall bias. Participating farmers were identified some 6–10 months after the fires ended as they were not recalling answers to questions correctly (information bias). It is also possible that some farms were not identified as veterinarians may not have recalled them (selection bias). It is also possible that some selected controls were not representative of the exposure in the source population. Whilst the majority of controls were random, with risk‐based sampling enabling good control selection, there was some limited controls convenience sampled in Victoria. Whilst these were less than 10% of total farms, these may have introduced some unknown biases into the results of this study.

In conclusion, this is the first systematic study of risk factors for bushfire injuries in livestock reported internationally. It reveals there are some important steps farmers can take to protect livestock. This included advanced planning and some advanced firefighting actions such as back burning although these should be cautiously applied given the legal and safety ramifications of such activities. Fire‐fighting authorities were protective and are an important resource for farmers, although the considerable skills of farmers can also be used by firefighting authorities. Enterprises with mixed sheep and beef production were more susceptible to bushfire injury than beef cattle or dairy enterprises, but further research is required to understand why this is the case.

## Conflicts of interest and sources of funding

The authors report no conflict of interest. This research was funded by Meat & Livestock Australia and the Commonwealth Government of Australia.

## Supporting information


**Appendix S1** Supporting Information.
