# Supplementary material for: The black summer bushfires: impacts and risk factors for livestock bushfire injury in south‐eastern Australia
Source: Aust Vet J. 2022 May 5;100(7):306–17. doi: 10.1111/avj.13165 (PMC9546107; doi:10.1111/avj.13165)
Supplement: Supplementary file 1 — Appendix S1 Supporting Information. [file AVJ-100-306-s001.docx]

# **Supporting information**

This supporting includes two sections, (1) some descriptive information and the (2) questionnaire survey of farmers.

# **Descriptive information**

# Figures illustrating comparability of cases and controls


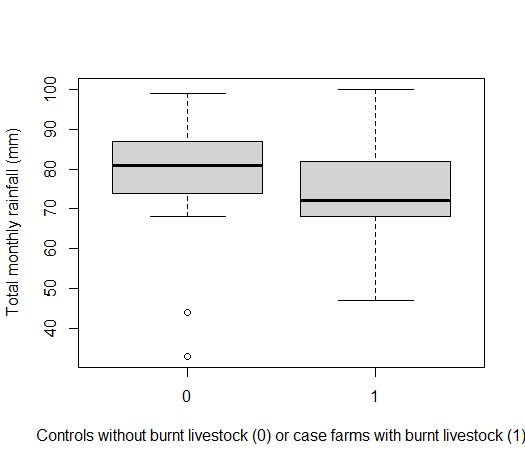


Figure S1 Average of monthly total rainfall (mm) for each farm for the two months prior to November 2019 for cases and controls.


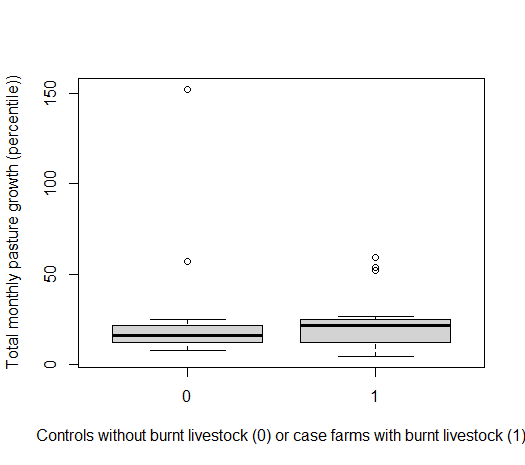


Figure S2 Monthly pasture growth (percentiles) for the two months preceding November 2019 for cases and controls


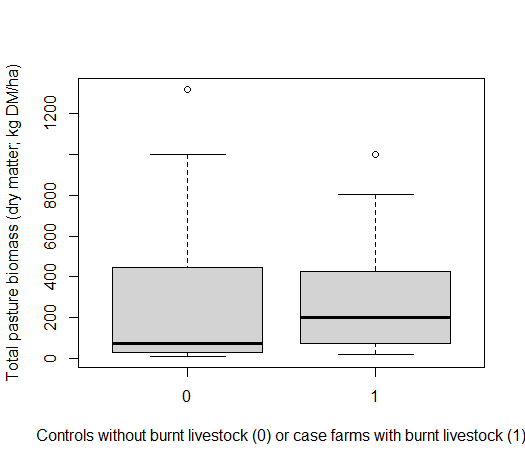


Figure S3 Total pasture biomass (dry matter as kg/ha) for the two months preceding November 2019 for cases and controls

# Descriptive statistics examining uncontrolled relationships between risk factors and cases and controls

Table S1 Un-controlled relationships between case farms and various explanatory variables

| **Farm enterprise or production type** | | | | | | | |
| --- | --- | --- | --- | --- | --- | --- | --- |
|  | Beef cattle | | | Beef cattle and Sheep | | Dairy | |
| No burnt livestock | 20 | | | 1 | | 4 | |
| Burnt livestock | 12 | | | 8 | | 1 | |
| Proportion burnt | 38% | | | 89% | | 20% | |
| **Farm Management (stocking rate perception)** | | | | | | | |
|  | Conservative | | | Medium | | High | |
| No burnt livestock | 12 | | | 6 | | 7 | |
| Burnt livestock | 8 | | | 8 | | 5 | |
| Proportion burnt | 40% | | | 57% | | 42% | |
| **Farm Management (Grazing management)** | | | | | | | |
|  | Set stocking and rotational | | | Rotational | | Set stocking | |
| No burnt livestock | 5 | | | 15 | | 5 | |
| Burnt livestock | 2 | | | 14 | | 5 | |
| Proportion burnt | 29% | | | 48% | | 50% | |
| **Farm Preparation (Removal of trees in paddocks)** | | | | | | | |
|  | No | | | Yes | | | |
| No burnt livestock | 16 | | | 9 | | | |
| Burnt livestock | 17 | | | 4 | | | |
| Proportion burnt | 52% | | | 31% | | | |
| **Preparation (Did you graze down a refuge paddock?)** | | | | | | | |
|  | No | | | Yes | | | |
| No burnt livestock | 9 | | | 12 | | | |
| Burnt livestock | 9 | | | 16 | | | |
| Proportion burnt | 50% | | | 43% | | | |
| **Preparation (Did you have a fire plan?)** | | | | | | | |
|  | No | | | Yes | | | |
| No burnt livestock | 2 | | | 23 | | | |
| Burnt livestock | 7 | | | 14 | | | |
| Proportion burnt | 78% | | | 38% | | | |
| **Preparation (How many fire-fighting units* did you have?)** | | | | | | | |
|  | 0 | | | 1-2 | | +2 | |
| No burnt livestock | 5 | | | 13 | | 7 | |
| Burnt livestock | 7 | | | 13 | | 1 | |
| Proportion burnt | 58% | | | 50% | | 13% | |
| **Response (Did you move stock on farm in response to fire?)** | | | | | | | |
|  | No | | | Yes | | | |
| No burnt livestock | 7 | | | 18 | | | |
| Burnt livestock | 6 | | | 15 | | | |
| Proportion burnt | 46% | | | 45% | | | |
| **Response (Did you move stock from farm in response to fire?)** | | | | | | | |
|  | No | | | Yes | | | |
| No burnt livestock | 22 | | | 3 | | | |
| Burnt livestock | 20 | | | 1 | | | |
| Proportion burnt | 48% | | | 25% | | | |
| **Response (Did you install a firebreak?)** | | | | | | | |
|  | No | | | Yes | | | |
| No burnt livestock | 12 | | | 13 | | | |
| Burnt livestock | 13 | | | 8 | | | |
| Proportion burnt | 52% | | | 38% | | | |
| **Response (Did you stay and defend?)** | | | | | | | |
|  | No | | | Yes | | | |
| No burnt livestock | 7 | | | 18 | | | |
| Burnt livestock | 6 | | | 15 | | | |
| Proportion burnt | 46% | | | 45% | | | |
| **Response (Backburn?)** | | | | | | | |
|  | No | | | Yes | | | |
| No burnt livestock | 19 | | | 6 | | | |
| Burnt livestock | 20 | | | 1 | | | |
| Proportion burnt | 51% | | | 14% | | | |
| **Response (Attack fire with water)** | | | | | | | |
|  | No | | | Yes | | | |
| No burnt livestock | 6 | | | 19 | | | |
| Burnt livestock | 5 | | | 16 | | | |
| Proportion burnt | 45% | | | 46% | | | |
| **Response (Containment lines)** | | | | | | | |
|  | No | | | Yes | | | |
| No burnt livestock | 17 | | | 8 | | | |
| Burnt livestock | 16 | | | 5 | | | |
| Proportion burnt | 48% | | | 38% | | | |
| **Response (Cut fence)** | | | | | | | |
|  | No | | | Yes | | | |
| No burnt livestock | 18 | | | 7 | | | |
| Burnt livestock | 16 | | | 5 | | | |
| Proportion burnt | 47% | | | 42% | | | |
| **Wind direction at worst time** | | | | | | | |
|  | Westerly | Easterly | North | | South | |  |
| No burnt livestock | 20 | 3 | 0 | | 2 | |  |
| Burnt livestock | 15 | 3 | 3 | | 0 | |  |
| Proportion burnt | 43% | 50% | 100% | | 0% | |  |

*A fire-fighting unit is a mobile water tank, hose and fire-fighting pump

# **Questionnaire survey**

Livestock and Australian Bushfires: Affected Farmer Interviews (concurrent with on-farm sampling)

Start of Block: 0 Pre-interview checks

Q0.1 Has the Consent Form "Project: Health, welfare and biosecurity of livestock exposed to Australian bushfires: an on-farm case control study" been signed by the participant?


(If not, please get Consent Form signed prior to commencing interview)

- Yes (1)
- No (2)

Q0.2 What is the project unique farm identifier for this farm? 


For NSW use the LLS Holding Reference Number (HRN) 
For VIC identifier should be in the format: Surname_First6numbersofcontactphone (e.g. “Cowled_042085”)

________________________________________________________________

Q0.3 What are the longitude and latitude of the yards where the samples are being taken today? 


(If yards not on fire-affected block, take coordinates of house or another landmark that will be visible on google maps. Include as many decimal places as provided by the device used. )

|  | List details (1) |
| --- | --- |
| Longitude (e.g. 150) (1) |  |
| Latitude (e.g. -40) (2) |  |
| Landmark type (if not cattle yards) (3) |  |

Q0.t1 Please read the following text to the participant: 


"We're going to talk in detail about the fire on your property in this interview. You are allowed to take a break anytime, if you would like a break please interrupt me and say so. We can finish the interview early at any time for any reason and you don't have to tell me why. If you would like to stop at any point please interrupt me and say so. There are no negative consequences for you if we do not complete the whole interview. Do you have any questions before we begin?"

End of Block: 0 Pre-interview checks

Start of Block: 1 Bushfire presence or absence

Q1.t1 Please read the following text to the participant: 


"There are nine sections in this interview, some will be quite quick and some may take a bit longer. We'll start with the first section, about details of the fire that occurred on the property we are talking about today."

Q1.1a Do you own the property we are discussing today?

- Yes (1)
- No (2)

Display This Question:

If Q1.1a = No

Q1.1b Given you are not the property owner, what role do you have on the this property?

- Manager (1)
- Share-farmer (2)
- Other (specify below) (3)

Display This Question:

If Q1.1a = No

And Q1.1b = Other (specify below)

Q1.1c What other role do you have on this property?

________________________________________________________________

Display This Question:

If Q1.1a = No

Q1.1d Given you are not the property owner, do you own any of the stock on this property?

- Yes (1)
- No (2)

Display This Question:

If Q1.1a = No

And Q1.1d = Yes

Q1.1e What percentage of the stock on this property do you own?

________________________________________________________________

Q1.t2 The rest of the questions on this page relate to the 2019-20 fire season. In the 2019-20 fire season:

Q1.2 (In the 2019-20 fire season) Was there bushfire on your farm land? 


Bushfire is defined as an uncontrolled fire that occurs in forest, scrub, woodland, grassland or pasture.

- Yes (1)
- No (2)

| 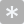 |
| --- |

Q1.3 (In the 2019-20 fire season) For how many days did bushfire burn on your farm?

________________________________________________________________

Display This Question:

If If (In the 2019-20 fire season)&nbsp;For how many days did bushfire burn on your farm? (enter a number e.g. 2)&nbsp; Text Response Is Not Equal to 1

| 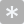 |
| --- |

Q1.4a What date did the fire start burning on your property, on the first day of bushfire (in the 2019-20 fire season)? (enter as dd/mm/yyyy)

________________________________________________________________

Display This Question:

If If (In the 2019-20 fire season)&nbsp;For how many days did bushfire burn on your farm? (enter a number e.g. 2)&nbsp; Text Response Is Not Equal to 1

Q1.4b What time on that date, to the nearest hour, did the fire start burning on your property, on the first day of bushfire (in the 2019-20 fire season)?

________________________________________________________________

| 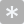 |
| --- |

Q1.5 What date was your 'worst fire day' (in the 2019-20 fire season)? (enter as dd/mm/yyyy)


For example, the worst day for fire on your farm, or the day the fire was a highest risk of damaging your farm. This should be the same day used when preparing the farm maps. 


This day is referred the 'study day' for the rest of the interview and will be the main focus for most of the survey.

________________________________________________________________

Q1.6 In what hour on that 'study day' did the fire start burning (or flare up if your property was already burning)?

________________________________________________________________

Q1.7a What were the main reason(s) fire ignited on (or nearest) your farm on the 'study day'? 
Can select one or multiple reasons.

- Main fire front reached farm (1)
- Spot fires (2)
- Persistent embers (3)
- Backburning (4)
- Lightning strike (5)
- An existing fire flared up (6)
- Other (specify below) (7)

Display This Question:

If Q1.7a = Other (specify below)

Q1.7b What was the other reason the fire ignited on the 'study day'?

________________________________________________________________

End of Block: 1 Bushfire presence or absence

Start of Block: 2 Fire severity or intensity

Q2.t1 All questions on this page relate to the 'study day' identified previously.

Q2.1 On the 'study day', did areas of pasture burn?

- Yes (1)
- No (2)

Display This Question:

If Q2.1 = Yes

Q2.2 What is your estimate of how fast the MAIN fire moved on pasture?

- Fast (>5 km/hr) (1)
- Medium (>1 to (2)
- Slow ( (3)

Display This Question:

If Q2.1 = Yes

Q2.3 What is your estimate of the height of the flame length on pasture (in metres)?

________________________________________________________________

Q2.4 On the 'study day', did wooded areas of the farm burn?

- Yes (1)
- No (2)

Display This Question:

If Q2.4 = Yes

Q2.5 What is your estimate of how fast the MAIN fire moved in wooded areas?

- Fast (>5 km/hr) (1)
- Medium (>1 to (2)
- Slow ( (3)

Display This Question:

If Q2.4 = Yes

Q2.6 What is your estimate of the height of the flame length in wooded areas (in metres)?

________________________________________________________________

Display This Question:

If Q2.4 = Yes

Q2.7 What is your estimate of the average depth of litter (fine fuel) on the ground in wooded areas (in cm)?

________________________________________________________________

Q2.8 How wide was the MAIN fire front?

- Spot fires or narrower than a paddock (i.e. (1)
- Broad front at least a paddock wide (i.e. >400m) (2)

End of Block: 2 Fire severity or intensity

Start of Block: 3 Fire history, topography and weather conditions

| 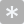 |
| --- |

Q3.1 What year did you start on this farm? 
(i.e. what year do you have detailed knowledge of the fire history of the property from?)

________________________________________________________________

| 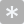 |
| --- |

Q3.2 To the best of your knowledge, in what year was the last bushfire on this farm prior to October 2019? 
(If not known, leave blank)

________________________________________________________________

Q3.3a From your observations, did the animals seek particular areas or topography on the farm in the face of the fire?

- Yes (1)
- No (2)

Display This Question:

If Q3.3a = Yes

Q3.3b What were the environmental features of the area that animals sought in the face of the fire?

________________________________________________________________

________________________________________________________________

________________________________________________________________

________________________________________________________________

________________________________________________________________

Q3.4 Think about areas of pasture that were near the fire but did not burn. Overall from your observations, what were the main common environmental features of those areas of pasture?

________________________________________________________________

________________________________________________________________

________________________________________________________________

________________________________________________________________

________________________________________________________________

Q3.5a
Think about the wind direction on the study day. Was the wind in a single direction or multiple directions?

- Single wind direction (1)
- Multiple wind directions (2)

Display This Question:

If Q3.5a = Single wind direction

Q3.5b What direction was the wind blowing from on the 'study day'?

________________________________________________________________

Display This Question:

If Q3.5a = Multiple wind directions

Q3.5c
Consider the 12 hours preceding the fire and the rest of the day on that 'study day'. What were the main directions the wind blew from on the 'study day', and what time did they change?

________________________________________________________________

________________________________________________________________

________________________________________________________________

________________________________________________________________

________________________________________________________________

End of Block: 3 Fire history, topography and weather conditions

Start of Block: 4 Farm type and management

Q4.t1 Please read the following text to the participant: 


"That's the first section complete. Do you want to take a short break, or is it ok for us to keep going?" 


Once participant indicates it's ok to keep going: 


"The next section of the interview is about details of the livestock on your farm and how you manage them. The questions relate to the stock you had on farm on the 'study day', just for the main block that was burnt."

Q4.1 Which types of production animals did you have on the block?

- Beef cattle (1)
- Dairy cattle (2)
- Sheep (3)
- Goats (4)
- Alpacas (5)

Display This Question:

If Q4.1 = Beef cattle

Q4.2a What type of beef enterprise did you have? (can select multiple)

- Self-replacing commercial (1)
- Self-replacing seedstock (2)
- Trading (3)
- Backgrounding (4)
- Agistment (5)
- Other (specify) (6)

Display This Question:

If Q4.2a = Other (specify)

Q4.2b What was the other type of beef enterprise?

________________________________________________________________

Display This Question:

If Q4.1 = Dairy cattle

Q4.2c What type of dairy enterprise did you have?

- Seasonal calving (1)
- Split calving (2)
- Year-round calving (3)

Display This Question:

If Q4.1 = Sheep

Q4.2d What type of sheep enterprise did you have?

- Self-replacing merino (1)
- Self-replacing prime lamb (2)
- Terminal sire to merino (breeding first cross ewes or terminal lambs) (3)
- Terminal sire to first cross ewes (4)
- Stud (5)
- All wethers (6)
- Trading e.g. trade lambs (7)
- Other (8)

Display This Question:

If Q4.2d = Other

Q4.2e What was the other type of sheep enterprise?

________________________________________________________________

Display This Question:

If Q4.1 = Beef cattle

Or Q4.1 = Dairy cattle

Q4.3a How many cattle did you have in each of the following groups on the 'study day' on this block?

|  | Number of animals (1) |
| --- | --- |
| Unweaned calves (1) |  |
| Weaned calves (up to 12 months) (2) |  |
| Yearlings & heifers (12 months to 2 years) (3) |  |
| Adult beef cows (>2 years) (4) |  |
| Adult dairy cows (>2 years) (5) |  |
| Adult bulls (>2 years) (6) |  |

Display This Question:

If Q4.1 = Sheep

Q4.3b How many sheep did you have in each of the following groups on the 'study day' on this block?

|  | Number of animals (1) |
| --- | --- |
| Unweaned lambs (1) |  |
| Weaners (up to 12 months) (2) |  |
| Hoggets & ewe lambs (12 months to 2 years) (3) |  |
| Adult ewes (>2 years) (4) |  |
| Adult rams (>2 years) (5) |  |
| Adult wethers (>2 years) (6) |  |

Display This Question:

If Q4.1 = Goats

Or Q4.1 = Alpacas

Q4.3c How many of these other species did you have in each of the following groups on the 'study day' on this block?

|  | Number of animals (1) |
| --- | --- |
| Q4.1 = Alpacas  Alpacas (total) (1) |  |
| Q4.1 = Goats  Dairy goats (total) (2) |  |
| Q4.1 = Goats  Non-dairy goats (total) (3) |  |

Q4.4 What is the typical weight of a dry (non-lactating) animal of each of the following types on your farm?

|  | Typical weight (kg) (1) |
| --- | --- |
| Q4.1 = Beef cattle  Dry beef cow (or steer) (1) |  |
| Q4.1 = Dairy cattle  Dry dairy cow (2) |  |
| Q4.1 = Sheep  Dry non-pregnant ewe (or wether) (3) |  |

Q4.5 What is the area of the block for each of the following purposes?

|  | Land area | Units (ha preferred) |
| --- | --- | --- |
|  | Enter area (1) |  |
| Total area (1) |  | ▼ Hectares (preferred) (1 ... Acres (2) |
| Area grazing land (includes fodder crops) (2) |  | ▼ Hectares (preferred) (1 ... Acres (2) |
| Area with commercial crops (3) |  | ▼ Hectares (preferred) (1 ... Acres (2) |
| Area for other purposes (not usable for livestock) (4) |  | ▼ Hectares (preferred) (1 ... Acres (2) |

Q4.6a How did you graze your stock in spring and summer 2019, prior to the fire in your region?

- Set stocking (1)
- Rotational grazing (2)
- Both set stocking and rotational grazing (3)

Display This Question:

If Q4.6a = Both set stocking and rotational grazing

Q4.6b What months was each grazing approach used?

|  | Month(s) (1) |
| --- | --- |
| Set stocking (1) |  |
| Rotational grazing (2) |  |

Q4.7 Consider the typical stocking rate on your farm and the landscape of this block. Overall, is your stocking rate:

- High (1)
- Medium (2)
- Conservative (3)

Q4.8a Do you irrigate pasture on this block?

- Yes (1)
- No (2)

Display This Question:

If Q4.8a = Yes

Q4.8b What proportion and time of year is the block irrigated?

|  | Proportion of pasture irrigated (1) | Months of year when irrigation occurs (2) |
| --- | --- | --- |
| For this block: (1) |  |  |

Q4.9 What was the average body condition score of your animals on the 'study day' and now? 
(Use the reference charts provided as guidance, score from 1 to 5. Estimate to nearest 0.5 of a condition score)

|  | Average body condition score on 'study day' (1) | Average body condition score now (2) |
| --- | --- | --- |
| If How many cattle did you have in each of the following groups on the 'study day' on this block?&nbsp; Adult beef cows (>2 years) - Number of animals Is Greater Than 0  And Q4.1 = Beef cattle  Adult beef cows (1) |  |  |
| Q4.1 = Beef cattle  Typical beef cattle (2) |  |  |
| Q4.1 = Sheep  And And How many sheep did you have in each of the following groups on the 'study day' on this block?&nbsp; Adult ewes (>2 years) - Number of animals Is Greater Than 0  Ewes (3) |  |  |
| Q4.1 = Sheep  And And How many sheep did you have in each of the following groups on the 'study day' on this block?&nbsp; Adult ewes (>2 years) - Number of animals Is Less Than 1  Or Or How many sheep did you have in each of the following groups on the 'study day' on this block?&nbsp; Adult ewes (>2 years) - Number of animals Is Empty  Typical sheep (4) |  |  |

Display This Question:

If Q4.1 = Beef cattle

Q4.9t

Display This Question:

If Q4.1 = Sheep

Q246

Q11.1a Were your stock receiving supplementary feed in spring and summer 2019, prior to the 'study day'?

- Yes (1)
- No (2)

Display This Question:

If Q11.1a = Yes

Q11.1b Which of these supplementary feeds were provided to stock in spring and summer 2019, prior to the 'study day'?

- Grain (1)
- Pellet (2)
- Hay/Roughage (includes silage) (3)
- Other (4)

End of Block: 4 Farm type and management

Start of Block: 5 Fire preparation

Q5.t1 Please read the following text to the participant: 


"That's the end of that section. The next section of the interview is about what you did to prepare before the fire on the study day. Any time you need a break, please interrupt me and we can take a break."

Q5.1 Do you routinely remove the following types of vegetation for fire preparedness: 
(remove includes clearing, thinning, weed control, etc.)

|  |  |
| --- | --- |
| Large trees (1) | ▼ Yes, routinely removed (1) ... No routine removal (2) |
| Smaller weeds and/or leaf litter (2) | ▼ Yes, routinely removed (1) ... No routine removal (2) |

Q5.2 Did you remove vegetation between August 2019 and the 2019-20 fires?

- Yes (1)
- No (2)

Q5.3 Did you purposefully graze down refuge paddocks to shelter stock in advance of the fire?

- Yes (1)
- No (2)

Q5.4a Did you have any firebreaks installed prior to the fire on the 'study day'?

- Yes (1)
- No (2)

Display This Question:

If Q5.4a = Yes

Q5.4b What kind of firebreaks were installed?

- Temporary firebreaks (1)
- Permanent firebreaks (2)

Q5.5 Did you have access to large volumes of water suitable for firefighting?

- Yes (1)
- No (2)

Q5.6 Did you have a fire plan in place at the time of the fires?

- Yes (1)
- No (2)

Q5.7 Did you plan on staying or going in the event of a fire in 2019/20? Was this what you did?

|  | Stay on-farm (1) | Go (2) |
| --- | --- | --- |
| Planned action (1) |  |  |
| Actual action (2) |  |  |

Q5.8a Did you have firefighting units on the farm? 
A unit is a mobile water tank, firefighting pump and hose.

- Yes (1)
- No (2)

Display This Question:

If Q5.8a = Yes

Q5.8b How many firefighting units did you have?

________________________________________________________________

Q5.9a Did you move stock between paddocks on-farm in advance of the fire or in response to the fire? (i.e. to protect them)

- Yes (1)
- No (2)

Display This Question:

If Q5.9a = Yes

Q5.9b Which paddock(s) did you move stock out of? (paddock name as listed on your paddock map)

________________________________________________________________

Display This Question:

If Q5.9a = Yes

Q5.9c Which paddock(s) did you move stock into? (paddock name as listed on your paddock map)

________________________________________________________________

Q5.10 Did you move stock off-farm as part of fire preparation?

- Yes (1)
- No (2)

Q5.11 In general, do you rely on the RFS/CFA for fire response?

- Yes (1)
- No (2)

Q5.12 Are there any other aspects of your fire preparations that you think are important that we haven’t discussed yet?

________________________________________________________________

________________________________________________________________

________________________________________________________________

________________________________________________________________

________________________________________________________________

End of Block: 5 Fire preparation

Start of Block: 6 Fire response

Q6.t1 Please read the following text to the participant: 


"That's the end of that section. The next section of the interview is about what you did to respond on that worse fire day that we're calling the 'study day'."  


Check in if the participant needs a break as required.

Q6.1 Did you stay and defend the farm during the fire?

- Yes (1)
- No (2)

Display This Question:

If Q6.1 = Yes

Q6.2 How many farm firefighting personnel were available during the fire (on average across the day)?

- One (1) person only (1)
- 2-3 people (2)
- 4-5 people (3)
- 6 or more people (4)

Display This Question:

If Q6.1 = Yes

Q6.3a Did farm firefighting personnel fire have personal protective equipment?
(for example, face masks, goggles, fire resistant clothing)

- Yes (1)
- No (2)

Display This Question:

If Q6.3a = Yes

Q6.3b What personal protective equipment did those people have?

________________________________________________________________

________________________________________________________________

________________________________________________________________

________________________________________________________________

________________________________________________________________

Display This Question:

If Q6.1 = Yes

Q6.4 How many farm firefighting personnel had firefighting training and/or experience?

- All (1)
- Some (2)
- None (3)

Q6.5 Did the farm firefighting personnel have communications equipment (such as radios)?

- Yes (1)
- No (2)

Q6.6 Did you receive assistance from RFS/CFA?

- Yes (1)
- No (2)

Q6.7 Was the fire accessible to firefighting (i.e. safe to approach) on the 'study day'?

- Yes (1)
- No (2)

Q6.8 Did you or the RFS/CFA make active efforts to fight the fire when your property was in fire?

- Yes (1)
- No (2)

Q6.9 Were your firefighting efforts directed towards:

|  | Yes (1) | No (2) |
| --- | --- | --- |
| House (1) |  |  |
| Other infrastructure (2) |  |  |
| Paddocks (3) |  |  |

Q6.10a Which (if any) of the following activities were undertaken immediately before (in the two weeks before) or
on the 'study day'?

- Bulldozed/ploughed fire breaks in advance of the fire (1)
- Back burning during the fire (2)
- Attacking fire with water (ground-based or aerial) (3)
- Blacking out containment lines at the edge of an active fire (with fire, chainsaws etc.) (4)
- Removal of ground fuel (e.g. leaves) (5)
- Targeted watering (6)
- Filled up all firefighting units (7)
- Cut fences (8)
- Other (specify in subsequent question) (9)
- None of these actions taken (10)

Display This Question:

If Q6.10a = Targeted watering

Q6.10b For the targeted watering, was this:

- Just around house (1)
- House and other places/areas on farm (2)
- Just other places/areas on farm (3)

Display This Question:

If Q6.10a = Cut fences

Q6.10b For cut fences, which paddocks were those fences between? (please list as paddock pairs or paddock name/laneway, using paddock names as listed on your paddock map)

________________________________________________________________

Display This Question:

If Q6.10a = Other (specify in subsequent question)

Q6.10c What other preparation actions were taken in the two weeks before the 'study day'?

________________________________________________________________

________________________________________________________________

________________________________________________________________

________________________________________________________________

________________________________________________________________

Q6.11a Did you lose any of the following farm infrastructure:

- House (1)
- Sheds (includes shearing shed if relevant) (2)
- Equipment/Machinery (3)
- Cattle yards (4)
- Sheep yards (5)
- Fences (6)
- Stored feed (7)
- Windbreaks (8)
- Other (specify in subsequent question, includes water tanks) (9)
- No infrastructure lost (10)

Display This Question:

If Q6.11a = Fences

Q6.11b Concerning fences lost, were these boundary or internal fences?

- Just boundary (1)
- Just internal (2)
- Both boundary and internal (3)

Display This Question:

If Q6.11b = Just boundary

Or Q6.11b = Both boundary and internal

Q6.11c What proportion (percentage) of your total boundary fencing was lost?

________________________________________________________________

Display This Question:

If Q6.11b = Just internal

Or Q6.11b = Both boundary and internal

Q6.11d What proportion (percentage) of your total internal fencing was lost?

________________________________________________________________

Display This Question:

If Q6.11a = Stored feed

Q6.10e What type of stored feed was lost?

________________________________________________________________

Display This Question:

If Q6.11a = Other (specify in subsequent question, includes water tanks)

Q6.11f What other infrastructure was lost?

________________________________________________________________

________________________________________________________________

________________________________________________________________

________________________________________________________________

________________________________________________________________

Q6.12 Are there any other aspects of your fire response that you think are important that we haven’t discussed yet?

________________________________________________________________

________________________________________________________________

________________________________________________________________

________________________________________________________________

________________________________________________________________

End of Block: 6 Fire response

Start of Block: 7 Fire recovery

Q7.t1 Please read the following text to the participant: 


"That's the end of that section. The next section of the interview is about what you did to recover after the study day."  


Check in if the participant needs a break as required.

Q7.1a Within the first week after the 'study day', which of the following activities did you undertake?

- Providing supplementary feed (1)
- Sold animals to salvage slaughter (2)
- Sold animals other than to salvage slaughter (e.g. to saleyards or to other producers) (3)
- Undertook activities to secure stock (e.g. emergency fencing) (4)
- Transported animals for agistment (5)
- Transported animals to another farm block (other than agistment) (6)
- Other (specify in subsequent question) (7)
- None of these activities undertaken (8)

Display This Question:

If Q7.1a = Providing supplementary feed

Q7.1b In the first week, to which animals and by what method did you provide supplementary feed?

|  | Provided supplementary feed | | How feed supplied | |
| --- | --- | --- | --- | --- |
|  | Yes (1) | No (2) | In paddock (1) | In feedlot/containment type area (2) |
| Beef cattle (1) |  |  |  |  |
| Dairy cattle (2) |  |  |  |  |
| Sheep (3) |  |  |  |  |

Display This Question:

If Q7.1a = Other (specify in subsequent question)

Q7.1c What other activities (related to your livestock enterprise) did you undertake in the first week after the 'study day'?

________________________________________________________________

________________________________________________________________

________________________________________________________________

________________________________________________________________

________________________________________________________________

Q7.2a After the first week and up to 6 months after the 'study day', which of the following activities related to livestock did you undertake?

- Providing supplementary feed (1)
- Transported animals for agistment (5)
- Transported animals to another farm block (other than agistment) (6)
- Sell animals with intention to buy stock back in later (4)
- Bought animals in to replace those lost or sold (3)
- Other livestock-related activity (specify in subsequent question) (7)
- None of these activities undertaken (8)

Display This Question:

If Q7.2a = Providing supplementary feed

Q7.2b After the first week and up to 6 months later, to which animals and by what method did you provide supplementary feed after the first week?

|  | Provided supplementary feed | | How feed supplied | |
| --- | --- | --- | --- | --- |
|  | Yes (1) | No (2) | In paddock (1) | In feedlot/containment type area (2) |
| Beef cattle (1) |  |  |  |  |
| Dairy cattle (2) |  |  |  |  |
| Sheep (3) |  |  |  |  |

Display This Question:

If Q7.1a = Providing supplementary feed

Or Q7.2a = Providing supplementary feed

Q11.2a Which of these supplementary feeds were provided to stock after the 'study day' (including up to 6 months later)?

- Grain (1)
- Pellet (2)
- Hay/Roughage (includes silage) (3)
- Other (list below) (4)

Display This Question:

If Q11.2a = Grain

Q11.2b For the grain, what type of grain?

- Wheat (1)
- Barley (2)
- Oats (3)
- Lupins (4)
- Other (list below) (5)

Display This Question:

If Q11.2a = Hay/Roughage (includes silage)

Q11.2c For the roughage, what type of roughage?

- Hay (1)
- Silage (2)
- Straw (3)
- Other (list below) (4)

Display This Question:

If Q11.2a = Other (list below)

Or Q11.2b = Other (list below)

Or Q11.2c = Other (list below)

Q11.2d What was the other type(s) of feed/(grain)/(roughage)

________________________________________________________________

Display This Question:

If Q7.2a = Sell animals with intention to buy stock back in later

Q7.2c For the animals sold with intention to buy stock back in later, what was the reason behind this decision?

- Decision based on economics/budgeting (1)
- Feed could not be sourced (regardless of price) (2)
- Not prepared to feed stock (regardless of price) (3)
- Other reason or more detail (specify in subsequent question) (4)

Display This Question:

If Q7.2c = Other reason or more detail (specify in subsequent question)

Q7.2d What was the other reason stock sold with the intention to buy back in later?

________________________________________________________________

________________________________________________________________

________________________________________________________________

________________________________________________________________

________________________________________________________________

Q7.3a After the first week and up to 6 months after the 'study day', which of the following activities related to pasture recovery did you undertake?

- Nitrogen application (once rain came with intent to grow more feed) (1)
- Oversow with annual pasture (2)
- Sow fodder crop (3)
- Other pasture recovery activity (specify in subsequent question) (4)
- No pasture recovery activity taken (5)

Display This Question:

If Q7.3a = Other pasture recovery activity (specify in subsequent question)

Q7.3b What was the other pasture recovery activity undertaken?

________________________________________________________________

________________________________________________________________

________________________________________________________________

________________________________________________________________

________________________________________________________________

Q7.4a Will you need to re-sow pastures?

- Yes (1)
- No (2)

Display This Question:

If Q7.4a = Yes

Q7.4b Which paddocks need to be re-sown? (paddock names as listed on your paddock map)

________________________________________________________________

________________________________________________________________

________________________________________________________________

________________________________________________________________

________________________________________________________________

Q7.5a Did you seek professional advice when deciding on recovery strategies?

- Yes (1)
- No (2)

Display This Question:

If Q7.5a = Yes

Q7.5b What type of professional adviser did you seek recovery strategy advice from?

________________________________________________________________

Q7.6 What is your estimate of the total cost of fire on your farm (in the 2019-20 fire season)?

________________________________________________________________

Q7.7 Have you changed your fire plan since this fire? If so, how?

________________________________________________________________

________________________________________________________________

________________________________________________________________

________________________________________________________________

________________________________________________________________

Q7.8 What lessons have you learnt from this fire season? What would you do differently next time?

________________________________________________________________

________________________________________________________________

________________________________________________________________

________________________________________________________________

________________________________________________________________

Q7.9 In the next sections, we’ll talk in detail about the effects on your livestock, including burns, health, reproduction and nutrition. Apart from those topics, are there any other aspects of your fire recovery that you think are important that we haven’t discussed yet?

________________________________________________________________

________________________________________________________________

________________________________________________________________

________________________________________________________________

________________________________________________________________

End of Block: 7 Fire recovery

Start of Block: 8 Burnt livestock

Q8.t1 Please read the following text to the participant: 


"That's the end of that section, we're more than halfway now. The next section of the interview is about the animal health and production effects you observed in your animals following the fire. We'll start with talking about burnt or singed animals, and then talk about other health and production effects you saw."


Check in if the participant needs a break as required.

Q8.1 Did you have any burnt livestock?

- Yes (1)
- No (2)

Q8.2 Did you have any singed livestock?

- Yes (1)
- No (2)

Display This Question:

If Q8.1 = Yes

Or Q8.2 = Yes

| 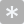 |
| --- |
